# TCAD Simulation of STI Depth and SiO_2_/Silicon Interface Trap Modulation Effects on Low-Frequency Noise in HZO-Based Nanosheet FETs

**DOI:** 10.3390/nano16040248

**Published:** 2026-02-13

**Authors:** Wonbok Lee, Jonghwan Lee

**Affiliations:** Department of System Semiconductor Engineering, Sangmyung University, Cheonan 31066, Republic of Korea; 2024d1018@sangmyung.kr

**Keywords:** technology, computer-aided design, nanosheet field-effect transistors, shallow trench isolation, interface trap density, low-frequency noise, hafnium–zirconium oxide

## Abstract

This study analyzed the low-frequency noise characteristics of nanosheet field-effect transistors (NSFETs) using technology computer-aided design (TCAD) simulations. In particular, the effects of shallow trench isolation (STI) depth and gate–insulator interface trap density on the device’s flicker noise power spectral density (PSD) were systematically evaluated. The simulation results show that as STI depth increases, excessive trap charges formed in the STI oxide can deplete or invert the substrate beneath the STI layer, reducing the threshold voltage of parasitic transistors and thereby increasing flicker noise. In contrast, the shallow STI structure’s trapped charge density was found to be lower than in thicker structures. Additionally, when an HfO_2_–ZrO_2_ (HZO)-based ferroelectric insulator is applied, improved gate–field control and reduced trap-induced noise are observed compared to HfO_2_. Optimization results indicate that the optimal noise performance is achieved with an STI depth of 3 nm and a SiO_2_/silicon interface trap density of 1 × 10^10^ eV^−1^cm^−2^. This study provides a design direction for low-noise NSFETs through structural (STI) and material (interface traps and HZO) optimization and is expected to contribute to the development of next-generation low-power, high-reliability logic devices.

## 1. Introduction

The development of transistors has had a profound influence on modern society, extending importance beyond their role as mere semiconductor components [1]. Transistors have evolved from metal-oxide-semiconductor field-effect transistors (MOSFETs) to Fin FETs, and further to gate-all-around FETs (GAAFETs), enhancing efficiency in critical aspects such as cost, performance, and energy consumption [2,3]. In addition, FinFET and GAAFET are examples of adopting an architecture that enables scaling by introducing the first and second trigate controls, respectively [4]. However, excessive scaling of FETs leads to short-channel effects (SCEs), resulting in an increased gate leakage current as the gate’s ability to control drain current diminishes [5]. Among the structures designed to suppress SCEs, nanosheet FETs (NSFETs), a type of GAAFET, exhibit improved performance compared to nanowire FETs (NWFETs) while also enabling vertical stacking and providing a larger channel area [6].

Ferroelectric field-effect transistors (FeFETs) have been extensively investigated for various memory-oriented applications due to their excellent scalability, energy efficiency, and compatibility with CMOS processes [7]. In NSFETs, HfO_2_, a fluorite-structure ferroelectric (FF) thin film, is used as the gate oxide, which influences device performance [8]. The ferroelectric (FE) properties of doped HfO_2_ allow further downscaling and low-power operation, demonstrating excellent performance in flash-like nonvolatile memory structures with superior process compatibility and high-speed operation [9,10]. Among HfO2-based films, FE HfZrO_2_(HZO) offers a low thermal budget, high remanent polarization, excellent endurance compared to other dopants, and it has the advantage of having a similar atomic radius and lattice parameters to HF [11,12].

Low-frequency noise is a powerful technique for evaluating the quality of oxide–semiconductor interfaces and channel layers [13]. In the low-frequency region, flicker noise and generation–recombination (G–R) noise are dominant. Flicker noise occurs in all semiconductor devices under bias conditions, while G–R noise arises from fluctuations in the number of carriers caused by the presence of G–R centers. These variations in carrier numbers lead to conductance fluctuations in the device. Additionally, FETs also exhibit shot noise, thermal noise, random telegraph signal noise, and burst noise [14,15].

Current research on shallow trench isolation (STI)-related noise mainly focuses on factors such as the distance between the STI and the channel, as well as channel width. However, few studies have directly analyzed the influence of variations in STI length on noise. Therefore, this study constructs a compact NSFET model based on HfO_2_ and HZO using technology computer-aided design (TCAD). Using this model, we investigate which material provides better noise performance and further explore how variations in charge density at the material interface and changes in STI width can be optimized to minimize noise.

## 2. NSFET Process and Simulation Setup

### 2.1. NSFET Physical Modeling with TCAD

**Figure 1:**
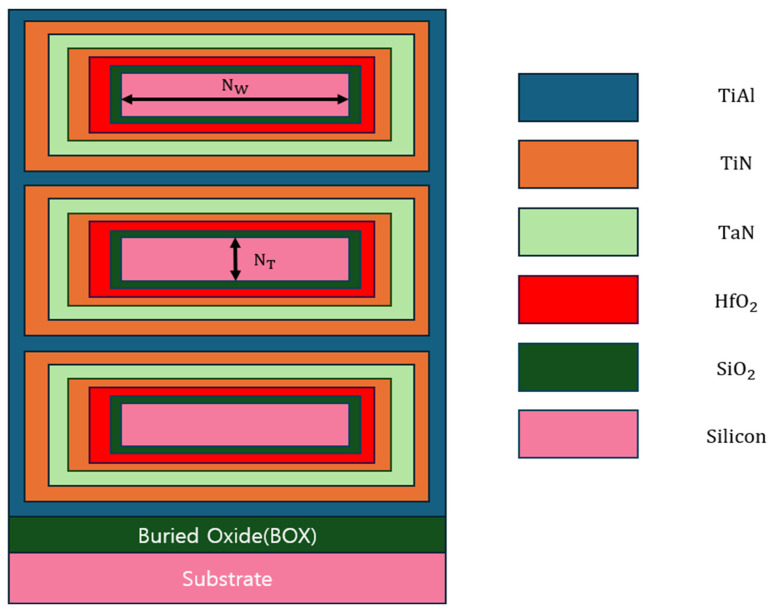
Gate stack of NSFET in the Y–Z plane.

**Figure 2:**
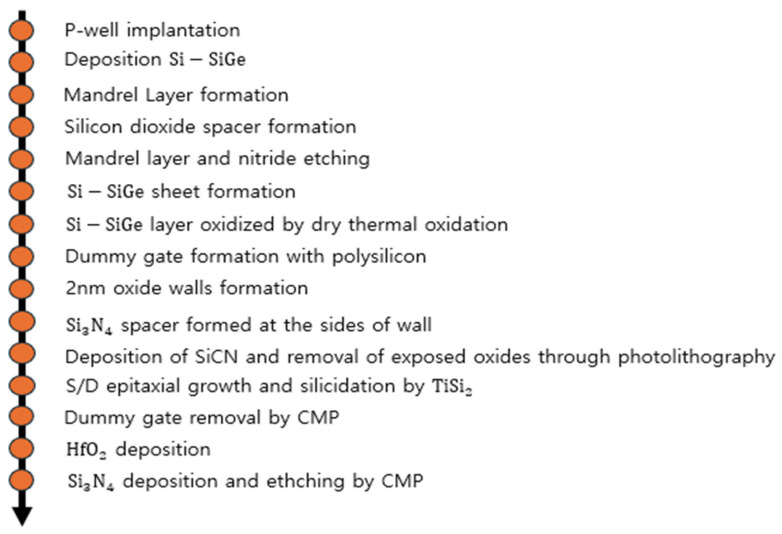
NSFET process.

**Figure 3:**
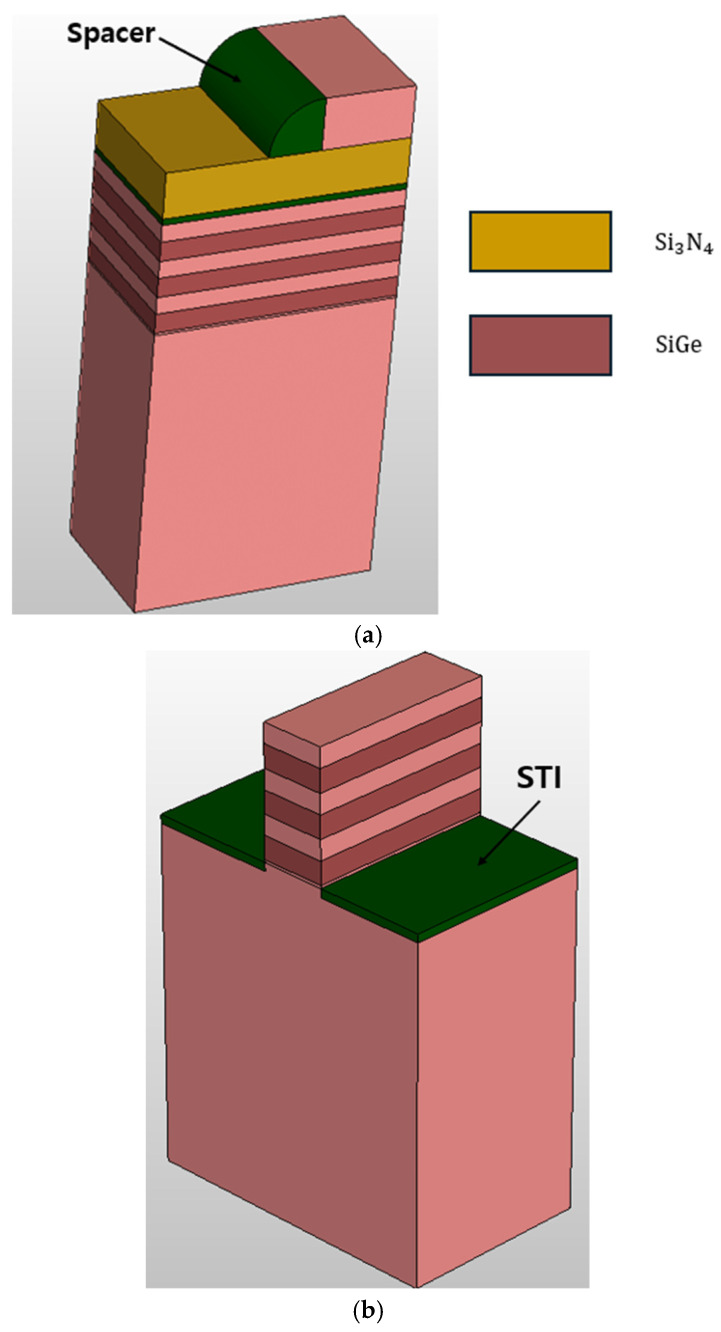
(**a**) SiO_2_ spacer formation. (**b**) Shallow-trench-isolation formation.

**Figure 4:**
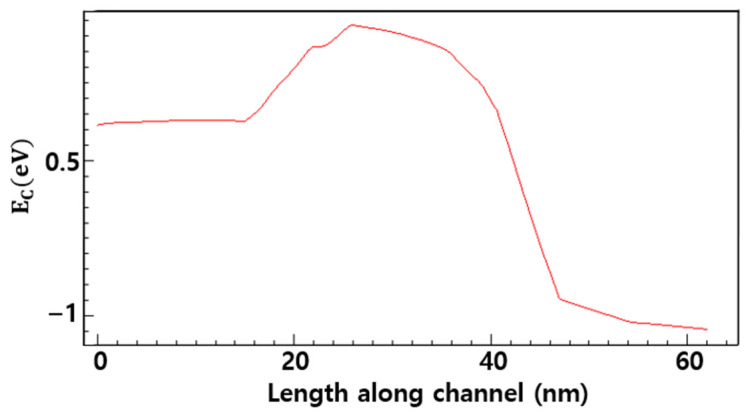
Conduction band energy profile at the channel surface of NSFET. (*V_DS_* = 0.7 V, V_GS_ = 1.0 V).

The process simulation of N-type NSFETs was conducted using Sentaurus TCAD Sprocess. Figure 1 shows the gate stack of the NSFET, and the process flow is illustrated in Figure 2. First, a boron dose was implanted onto the bulk wafer at an energy of 30 eV, followed by rapid thermal annealing at 1000 °C. Subsequently, three layers of silicon–germanium and three layers of silicon were alternately deposited on the silicon substrate. These layers were then covered with SiO_2_ (buffer oxide), silicon nitride (transfer layer), and silicon (mandrel layer). The mandrel layer was patterned through etching using photolithography. SiO_2_ spacers were formed through anisotropic etching, followed by isotropic overetching. Figure 3 confirms the formation of the SiO_2_ spacers. The mandrel layer was then etched, and the spacer served as a hard mask for etching the transfer layer. The residual nitride of the transfer layer acted as a hard mask for etching the nanosheet, and this nitride hard mask is a key element in sheet formation. To form the STI, before removing the remaining oxide through etching, the substrate region where silicon had been removed was filled with SiO_2_, which was then planarized using chemical mechanical planarization (CMP). Figure 4 confirms the formation of the STI. Next, a dummy gate was formed to mark the region where the channel would be located, and a thin oxide layer was grown on both sides of the dummy gate. To form the source and drain, epitaxial growth of boron-doped silicon was performed. Subsequently, a silicide process was conducted to reduce the contact resistance of the source and drain. All structures of the N-type NSFET were then covered with phosphosilicate glass (PSG), and CMP was used to remove the PSG above the dummy gate. A dielectric layer was then deposited to prevent breakdown and leakage current caused by quantum tunneling. A tungsten layer was deposited on the stack, 1 nm below the PSG level, using CMP. Between the PSG and tungsten layers, an additional dielectric was deposited and subsequently planarized using CMP. Finally, a cavity was created in the PSG through the self-aligned contact process. Table 1 lists the parameter values used for the N-type NSFET [16,17].

### 2.2. Device Characteristics of NSFET

**Figure 5:**
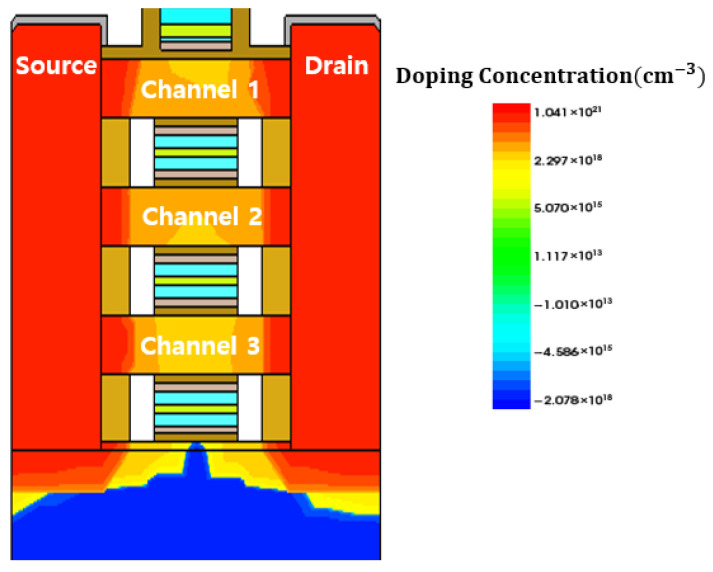
Doping concentration of N-type NSFET.

The device characteristics simulations were carried out under conditions of drain-to-source voltage (*V_DS_*) = 0.7 V and gate-to-source voltage (V_GS_) = 1.0 V with doped HfO_2_. Figure 4 shows the conduction-band energy profile of the NSFET in the channel region, and Figure 5 shows the NSFET doping concentration, allowing the distribution of dopants across various regions, such as the channel, source, and drain, to be visually confirmed.

**Figure 6:**
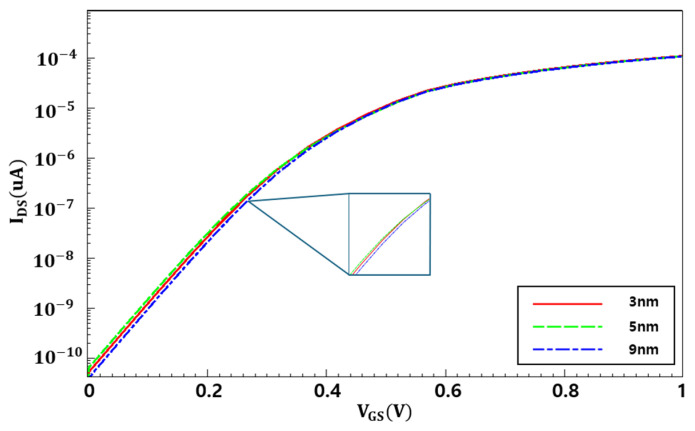
I-V characteristics of the N-type NSFET (*V_DS_* = 0.7 V, V_GS_ = 1.0 V).

Figure 6 shows the drain current (I_DS_)—V_GS_ characteristics of the N-type NSFET under varying STI depths at 3 nm, 5 nm, and 9 nm, which are used to extract various device parameters. The variation in I_DS_—V_GS_ characteristics with STI depth is associated with differences in compressive stress induced in the active region due to changes in the STI structure. As the STI depth varies, the compressive stress originating from the oxide to silicon is changed, which in turn affects carrier mobility and threshold-voltage characteristics, leading to changes in the drain current [18].

STI, a structural feature commonly implemented in MOSFETs for complementary metal-oxide-semiconductor (CMOS) processes, affects key device parameters, such as threshold voltage (Vth), transconductance, leakage current, and drain current [18]. Therefore, variations in STI also influence noise behavior. However, low-frequency noise (LFN) can change significantly even when the global DC transfer characteristics change only marginally, because flicker noise is more sensitive to local imperfections/trap activity along the current path [19]. In NWFETs, the FE layer acts as a negative capacitor, amplifying the surface potential beyond the applied gate voltage. The subthreshold swing (*SS*) is given by Equation (1), where *V_g_* denotes the gate voltage, *φ_s_* the surface potential at the Si-SiO_2_ interface, *I_d_* the drain-to-source current, *k* the Boltzmann constant, *q* the elementary charge, *C_s_* and *C_ins_* the capacitances of the semiconductor and insulator, and T the temperature [20].

(1) $$SS=\frac{\partial V_{g}}{\partial \varphi_{s}}\;\frac{\partial \varphi_{s}}{\partial {log}_{10}\left(I_{d}\right)}=\frac{kT}{q}\left(1+\frac{C_{s}}{C_{ins}}\right)\ln\left(10\right)$$

Drain-induced barrier lowering (DIBL), a key parameter indicating SCEs, is expressed by Equation (2) and is calculated from the difference between small drain voltage (*V_Dlin_*), *V_DS_* = 0.07 V, and large drain voltages (*V_Dsat_*), *V_DS_* = 0.7 V [21].

(2) $$DIBL=\frac{V_{th@V_{Dlin}}-V_{th@V_{Dsat}}}{V_{Dsat}-V_{Dlin}}$$

Table 2 lists the subthreshold swing (SS) and DIBL values according to variations in STI depth.

### 2.3. NSFET Physical Noise Mechanism

The simplest way to obtain flicker noise characteristics is to superpose various generation–recombination (G–R) noise spectra arising from the random trapping and emission of free carriers by centers with different lifetimes. This constitutes the basic concept of the McWhorter model, which can be expressed by Equation (3) [22].

(3) $$S^{1/f}\propto {\left(\Delta N\right)}^{2}\int_{\tau_{1}}^{\tau_{2}}\frac{1}{\tau }\frac{4\tau }{1+\omega \tau^{2}}\cdot d\tau ={\left(\Delta N\right)}^{2}\cdot \frac{1}{f}\;for\;\frac{1}{\tau_{2}}\ll \omega \ll \frac{1}{\tau_{1}}$$

where *τ*, **Δ*N***, and *ω* represent the time constant, fluctuation in the number of electrons in the channel, and angular frequency, respectively.

In silicon MOSFETs, low-frequency noise is dominated by flicker noise. Flicker noise is referred to as 1/*f* noise because its spectral density is inversely proportional to frequency [23]. Flicker noise originates from random charge capture (trapping) and emission (de-trapping) processes at oxide traps near the SiO_2_/silicon interface, leading to fluctuations in the channel carrier density [24,25]. These oxide traps dynamically exchange carriers with the channel, inducing fluctuations in the surface potential, which, in turn, modulate the channel carrier density and cause variations in the inversion charge density [26,27]. The current spectral density of flicker noise is expressed by Equation (4).

(4) $$S_{id,\frac{1}{f}}=(\frac{2}{F}+\frac{B}{F^{2}})\frac{q^{2}{I_{D}}^{2}N_{d}(E_{FN})}{{\epsilon_{ox}}^{2}WL^{\prime }\alpha f^{\eta }}$$

where $F=\sqrt{{E_{x}}^{2}+{E_{y}}^{2}+{E_{z}}^{2}}$ denote the electric field, B is a fixed constant, and *I_D_*, *L′*, and *N_d_* (*E_FN_*) represent the drain current, effective gate length, and trap density at the interface, respectively. The parameters *f*, *α*, and *ϵ_ox_* denote frequency, attenuation factor, and dielectric permittivity of the gate oxide, respectively. While Equation (4) follows a conventional trap-related flicker noise formalism, ferroelectric gate dielectrics such as HZO can exhibit additional dependence on polarization state and cycling history. Experimental studies on HZO-based ferroelectric vertical GAA FETs report that the measured LFN can be captured by carrier-number fluctuation (CNF) and mobility fluctuation (MF) formalisms through model fitting, supporting a CNF/MF-based framework as a first-order approach [28].

The generation–recombination (G–R) noise originates from local fluctuations in the number of free carriers caused by random transitions of charge carriers between different energy states within a semiconductor. Localized energy states, such as traps, deep donors, or Shockley–Read–Hall centers, stochastically capture and emit electrons and holes, leading to temporal variations in the mean value and variance of the free carrier number *N* [29]. The occurrence probability and characteristic lifetime of these capture–emission processes are governed by the relative energy conditions between the trap states and the energy levels occupied by the carriers, such that carrier number fluctuations are enhanced when transitions between energy states are energetically favorable. Consequently, these temporal fluctuations in the carrier population manifest as fluctuations in current and resistance, which are observed as generation–recombination noise [30,31]. Equation (5) represents the current spectral density of G–R noise.

(5) $$S_{id,GR}=\frac{N_{T}}{N^{2}}\frac{\tau }{1+4\pi^{2}f^{2}\tau^{2}}$$

where *N_T_*, *N*, and τ represent the trap count, number of carriers, and time constant, respectively.

Drain-current noise can be interpreted as an equivalent gate-voltage source applied at the input through the gate-coupling capacitance, analogous to amplifier noise models. In practice, because this voltage is fixed, the noise is not generated through changes in gate voltage. Instead, it arises from changes in oxide charge that cause variations in the surface potential tail. Additionally, noise due to mobility fluctuations occurs along the conduction path through which charge carriers move. The equivalent input gate voltage noise is considered a mathematical construction derived from drain-current noise using a transconductance (*g_m_*). Thus, the relationship between drain-current noise spectral density (*S_id_*) and gate voltage noise spectral density (*S_vg_*) is expressed by Equation (6) [32].

(6) $$S_{vg}=\frac{S_{id}}{{g_{m}}^{2}}$$

LFN was simulated using the built-in SDEVICE noise options with a consistent default parameter set [33]. Because the absolute LFN magnitude can depend on the selected noise-model parameters, this work emphasizes comparative trends obtained under an identical parameter set across STI depth splits.

## 3. TCAD Simulation of NSFET

### 3.1. HZO vs. HfO_2_ from a Noise Perspective

To verify the FE of HZO, a hysteretic loop is used to confirm polarization behavior. The Gibbs free energy (U–P) is based on the Landau–Khalatnikov (LK) equation. Equations (7) and (8) represent the Gibbs free energy, where *U*, *P*, and *E* denote energy, polarization, and electric field, respectively. The fitting parameters α, β, and γ use the values in Table 3. As the dielectric constant is inversely proportional to α, a reduction in α leads to an improvement in the dielectric constant. In this work, we adopted the parameters extracted by Lee, M.H., et al. [34].

(7) $$U=\alpha P^{2}+\beta P^{4}+\gamma P^{6}-E\cdot P$$

(8) $$E=2\alpha P^{2}+4\beta P^{3}+6\gamma P^{5}$$

**Figure 7:**
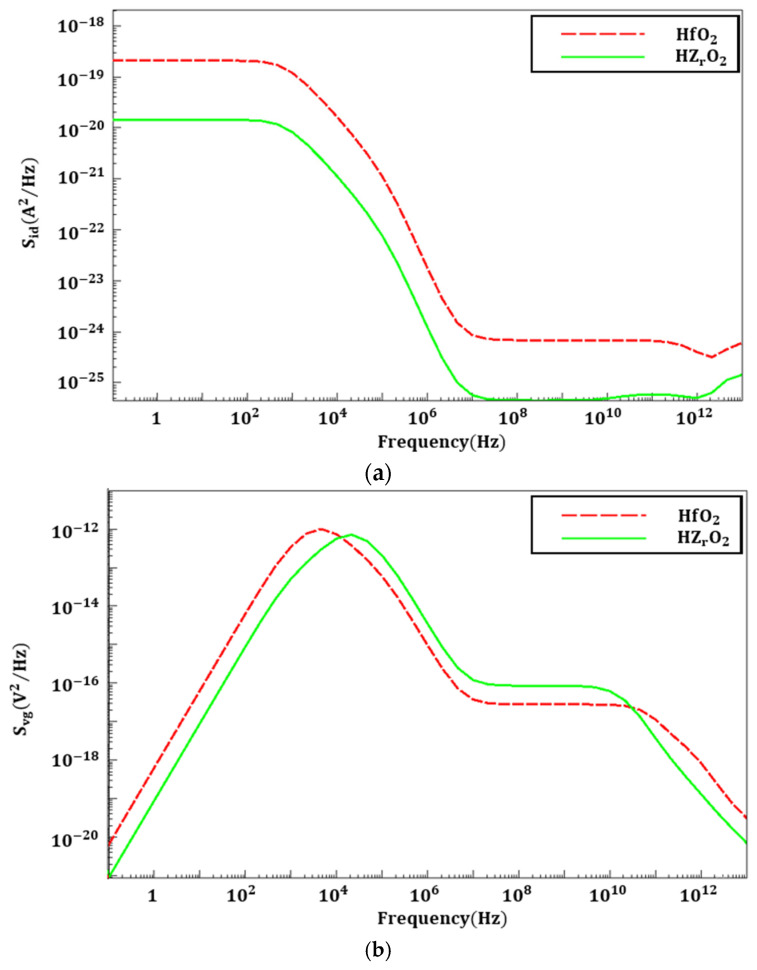
(**a**) S_id_ and (**b**) S_vg_ flicker noises of HZO and HfO_2_.

According to Maryam et al., adding elements such as ZrO_2_ to HfO_2_ reduces the oxide trap density, indicating that ZrO_2_ incorporation leads to noise reduction [35]. In this work, the HZO-related LFN behavior is discussed primarily within a trap-mediated flicker noise perspective, where the PSD is governed by carrier-number fluctuations associated with trapping/de-trapping activity in the gate dielectric and interface region [9]. This approach is supported by experimental LFN studies on ferroelectric-doped HfO_2_ gate dielectric performed under linear bias conditions, which report predominantly 1/*f*-like spectra governed by trapping and a lower PSD for the ferroelectric-doped HfO_2_ gate dielectric compared to the undoped HfO_2_ reference, consistent with a reduced trap density [9]. Accordingly, the observed HZO–HfO_2_ noise difference is attributed qualitatively to defect/trap engineering, without introducing an explicit polarization-fluctuation noise term. The simulation is conducted using the same physical model. In this simulation, the thickness of the HZO film is set to 5 nm. Previous studies have shown that even when the HZO film thickness is reduced to approximately 5 nm, a remanent polarization of about 10 µC/cm^2^ can be maintained, enabling saturated FE polarization at a low operating voltage of 1.0 V [36]. Figure 7 shows the S_id_ and S_vg_ of HfO_2_ and HZO under conditions where V_GS_ = 1.0 V, V_DS_ = 0.7 V, and the trap concentration of oxide is 1 × 10^10^ eV^−1^cm^−2^. The Gaussian distribution of the trap is expressed by *D_Gau_* = *N_0_*(*−*(*E − E_0_*)^2^/2*E_s_*^2^), where *N_0_* is a peak Gaussian trap distribution, and *E_0_* and *E_s_* denote its energy mean and energy standard deviation of the Gaussian distribution. The values considered for *E_0_* and *E_s_* are 0.1 eV and 0.1 eV, respectively [17]. By comparing S_id_ and S_vg_ for HfO_2_ and HZO, it can be confirmed that noise performance improves when ZrO_2_ is added to HfO_2_.

### 3.2. Simulation of Material Interface Trap Density

**Figure 8:**
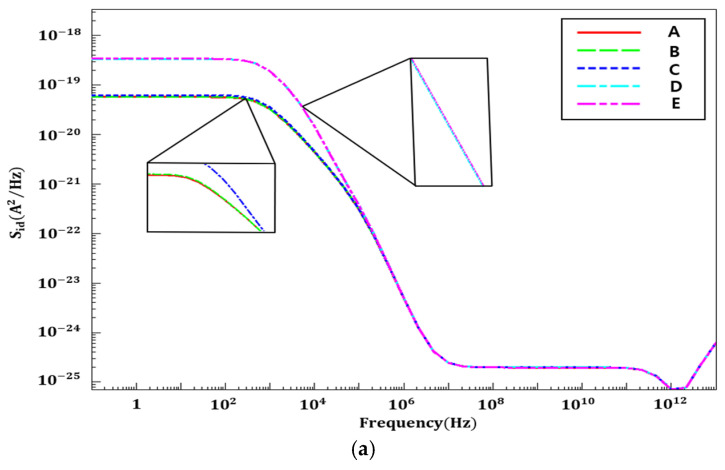

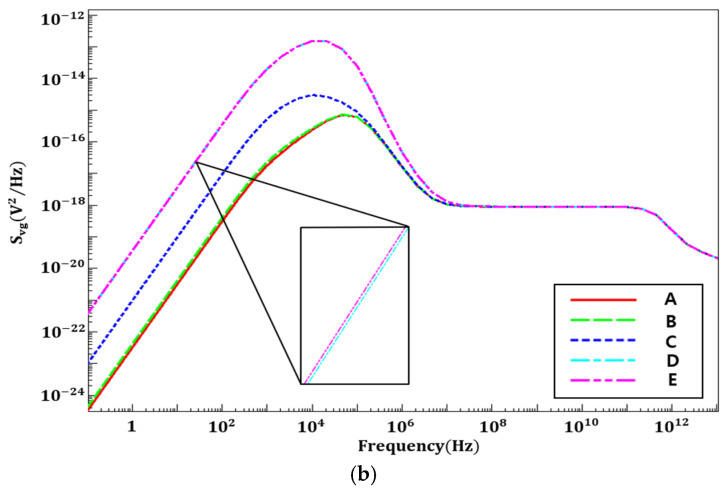
(**a**) S_id_ flicker noise. (**b**) S_vg_ flicker noise at different charge density values (SiO_2_/silicon interface trap densities = 1 × 10^10^ (A), 1 × 10^11^ (B), 1 × 10^12^ eV^−1^cm^−2^ (C), 1 × 10^13^ eV^−1^cm^−2^ (D), and 1 × 10^14^ eV^−1^cm^−2^ (E)).

The variation in noise with changes in trap density at the material interface (SiO_2_/silicon). The performance of HZO-doped NSFETs is simulated. As the number of oxide traps increases, electron trapping and de-trapping within the traps also increase, leading to a rise in total flicker noise [35,37]. Figure 8 shows the power spectral density (PSD) of drain current and gate voltage as a function of material interface charge density in HZO-based NSFETs. The interface trap density at the HZO/SiO_2_ interface is fixed at 1 × 10^10^ eV^−1^cm^−2^, while the trap densities at the SiO_2_/silicon interface are varied as follows: 1 × 10^10^ (A), 1 × 10^11^ (B), 1 × 10^12^ eV^−1^cm^−2^ (C), 1 × 10^13^ eV^−1^cm^−2^ (D), and 1 × 10^14^ eV^−1^cm^−2^ (E). The simulation results demonstrate that when the interface trap density at the SiO_2_/silicon interface is 1 × 10^10^ eV^−1^cm^−2^, HZO-doped NSFETs achieve better noise performance than other values.

### 3.3. STI Depth-Dependent Noise Characteristics

**Figure 9:**
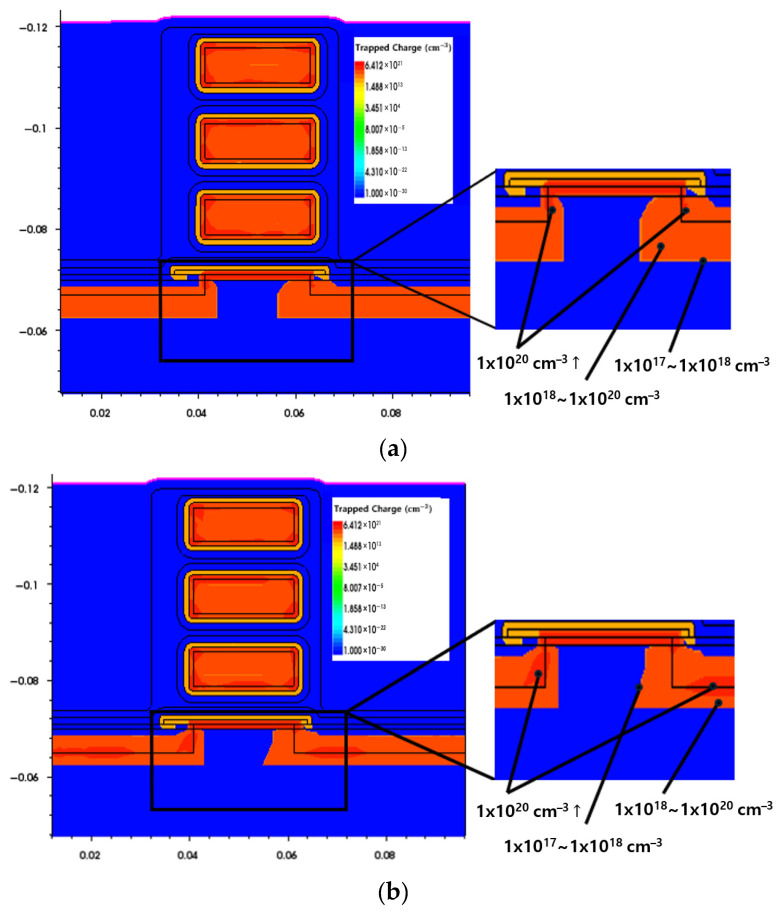

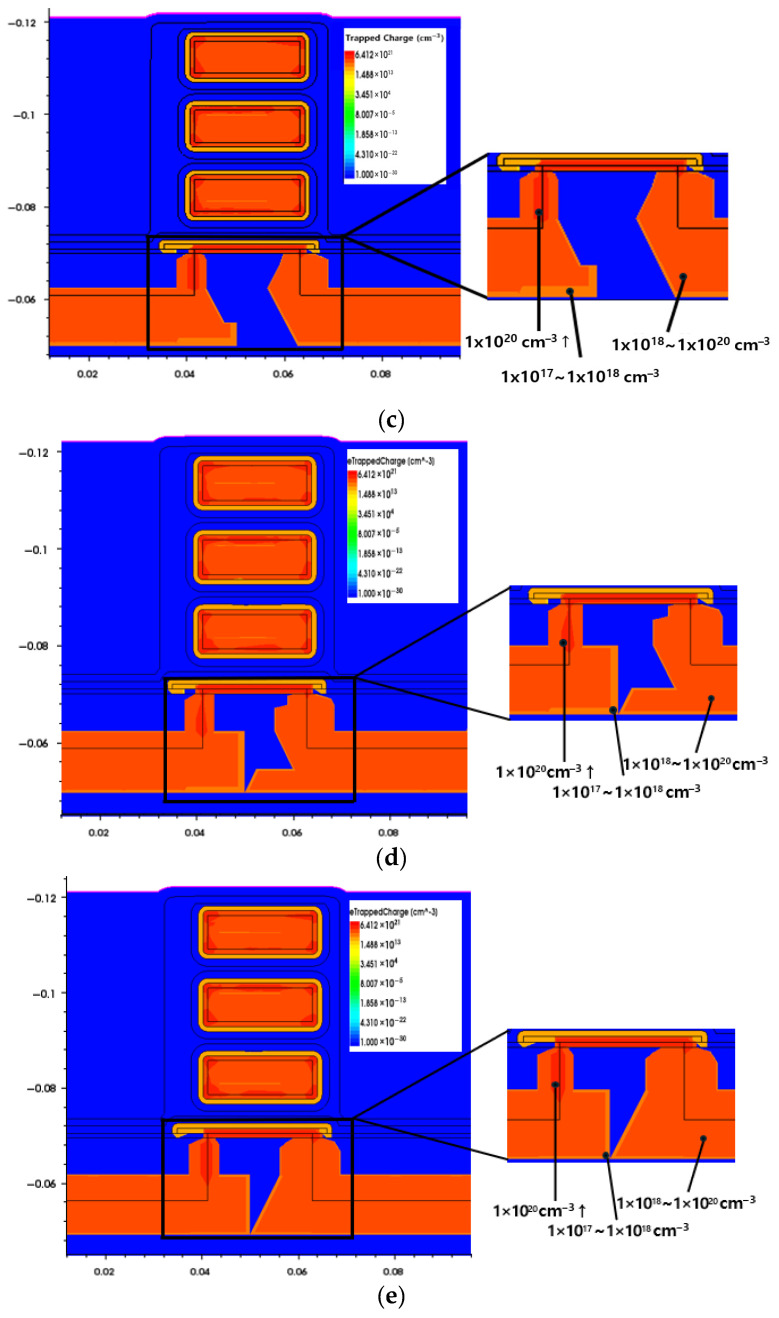
NSFET trapped charge density at STI depth change: (**a**) 3 nm, (**b**) 5 nm, (**c**) 9 nm, (**d**) 11 nm, and (**e**) 13 nm.

**Figure 10:**
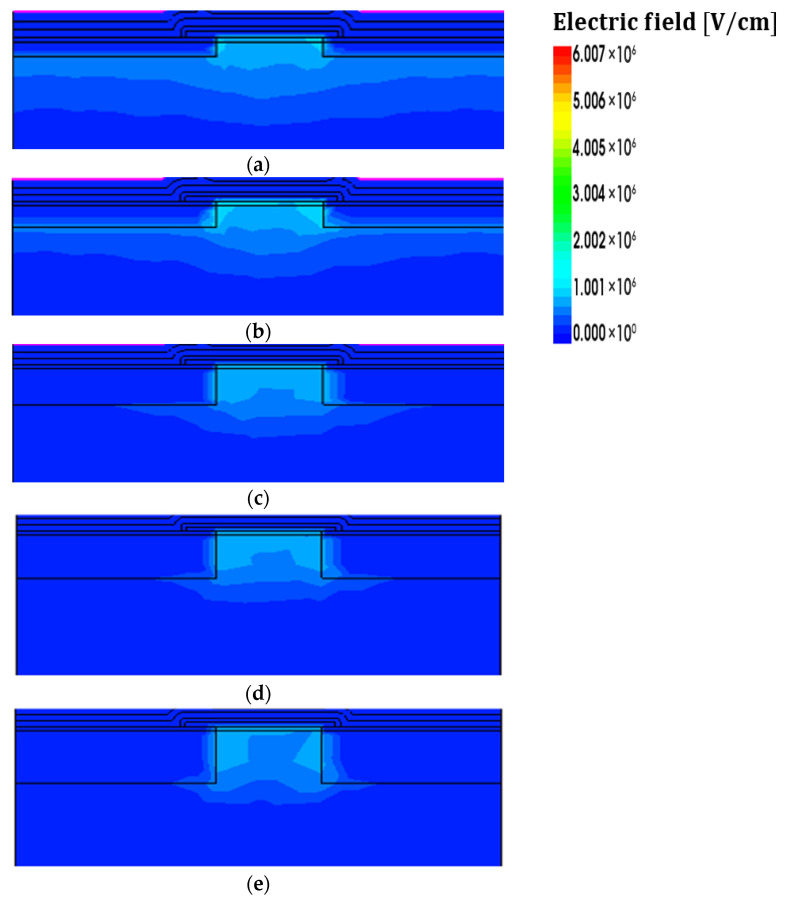
NSFET electric field distribution at STI depth change (**a**) 3 nm, (**b**) 5 nm, (**c**) 9 nm, (**d**) 11 nm, and (**e**) 13 nm.

The STI oxide can influence drain-to-source leakage current and off-state current behavior; therefore, excessively shallow STI structures may complicate the interpretation of the device’s electrical characteristics. Accordingly, simulations in this work were performed for STI depths of 3 nm and above [38]. This lower bound is motivated by isolation literature indicating that aggressive isolation scaling can increase leakage and degrade gate–oxide integrity, implying a practical trade-off beyond LFN minimization [39]. Hot-carrier reliability studies on STI-based/narrow-width devices also report enhanced degradation due to current crowding and electron trapping at the STI edge, suggesting reliability-aware constraints [40]. Accordingly, the STI = 3 nm recommendation is presented as a noise-driven optimum under these reliability-aware constraints. Figure 9 shows the distribution of charge traps near the channel as a function of STI depth. The trap charge density near the STI oxide ranges from approximately 1 × 10^17^ to 1 × 10^20^ cm^−3^ [41]. Research on noise with STI depth as an independent variable is limited. Therefore, this study investigates the noise characteristics by examining changes in trap charge distribution obtained from TCAD simulations. According to Rongbin et al. [42], excessive trap charges formed in the STI oxide can deplete or invert the substrate beneath the STI layer, reducing the threshold voltage of parasitic transistors and thereby increasing flicker noise. In addition, under higher electric fields, the enhanced carrier interaction with traps increases the fluctuation rate, which directly leads to the observed increase in flicker noise magnitude [43,44]. Figure 10 shows the electric field distribution of the NSFET for different STI depths of 3, 5, 9, 11, and 13 nm. As the STI depth increases, a clear enhancement of the electric field is observed near the STI/silicon interface, particularly around the STI corner region adjacent to the channel. This behavior indicates that increasing STI depth leads to a spatial redistribution of the electric field at the SiO_2_/silicon boundary, resulting in an expanded high-field region rather than a simple increase in peak field strength. The intensified electric field near the STI region is consistent with the increased trap charge density shown in Figure 9 and contributes to enhanced carrier trapping and de-trapping processes. Therefore, the simulated electric field redistribution provides a theoretical basis for the observed STI depth–dependent noise behavior. Based on this, it can be inferred that as STI depth increases, the PSD of the channel also increases, particularly if the interface trap density near the SiO_2_/silicon interface is high. In addition, TCAD results indicate that STI depth primarily modulates trap-related flicker noise via changes in trapped charge and local electric-field distribution, which is compatible with compact-model workflows where trap-governed LFN can be mapped to BSIM-CMG noise parameters via subthreshold and linear region fitting [45].

**Figure 11:**
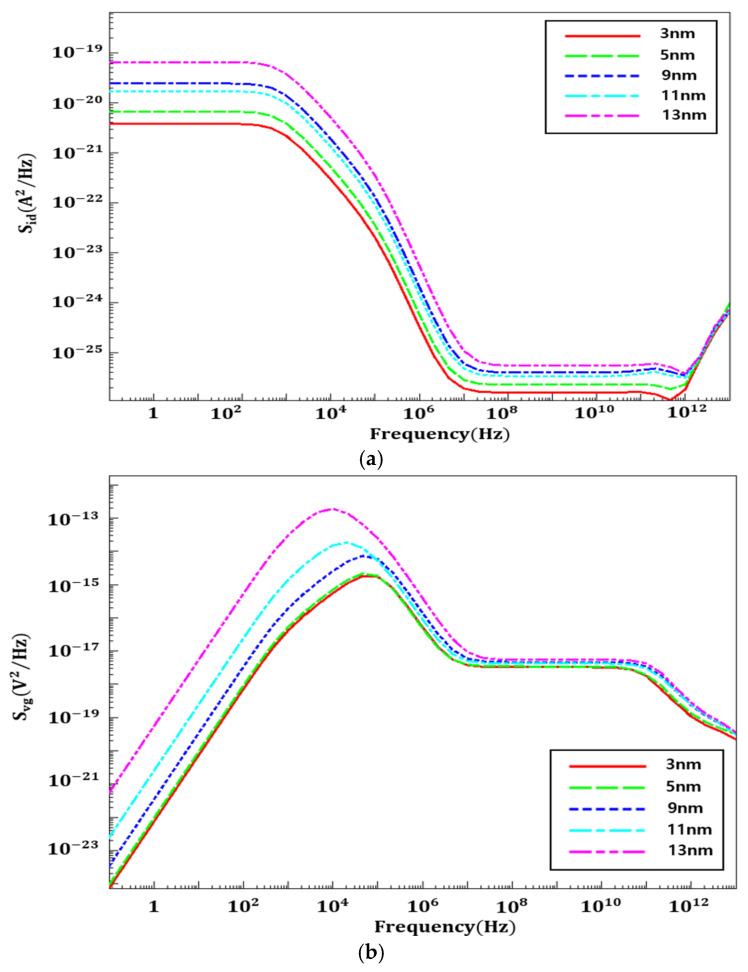
(**a**) S_id_ flicker noise and (**b**) S_vg_ flicker noise at different STI depths.

Figure 11 shows the drain current and gate voltage PSD characteristics for STI depths of 3, 5, 9, 11, and 13 nm. As illustrated, PSD values increase across the entire frequency range with increasing STI depth, consistent with the results inferred from trap charge distribution. In other words, deeper STI structures lead to more charge traps near the STI–channel interface, which enhances channel charge fluctuations and amplifies flicker noise.

## 4. NSFET Parameter Optimization

**Figure 12:**
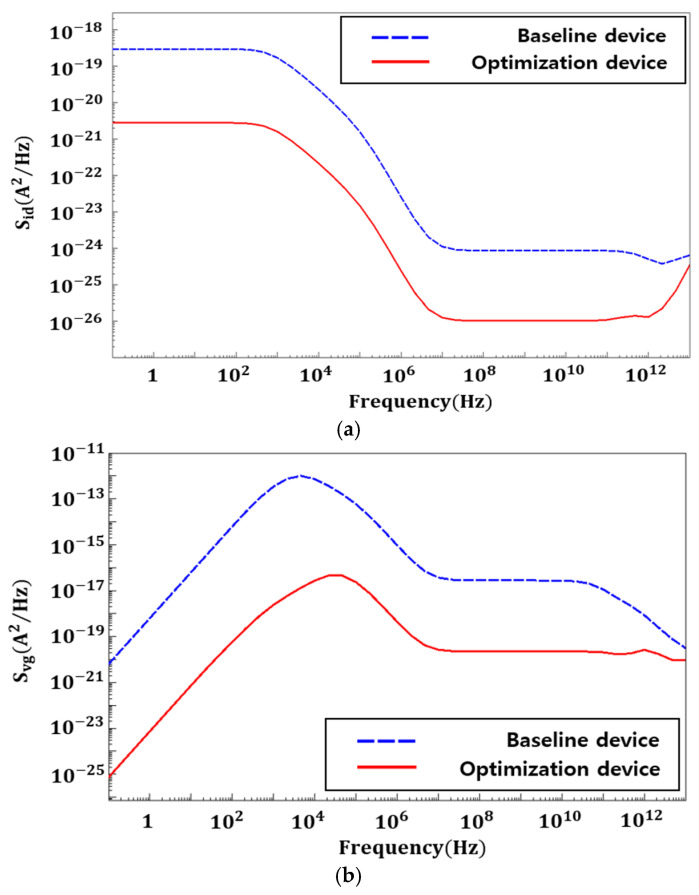
(**a**) S_id_ flicker noise at baseline device and optimization parameters. (**b**) S_vg_ flicker noise at baseline device and optimization parameters.

Based on the previous analyses, key structural and material parameters were optimized to improve the low-frequency noise performance of NSFETs. The baseline device in Figure 12 represents the PSD of an NSFET doped with HfO_2_ as the FE layer without STI formation, whereas the optimized device shows the PSD when the optimal material interface trap density and STI depth (3 nm)—from a noise perspective—are applied to an NSFET doped with HZO instead of HfO_2_. As STI depth increases, trap formation near the channel increases, leading to a corresponding rise in PSD. Furthermore, when the trap density at the SiO_2_/silicon interface is controlled at 1 × 10^10^ eV^−1^cm^−2^, trap-induced charge fluctuations decrease, resulting in reduced flicker noise. This improvement is attributed to enhanced interfacial quality of the gate insulator and suppression of charge-trapping paths. Therefore, it was confirmed that the combination of an HZO-based gate insulator with an STI depth of 3 nm provides the most stable and lowest-noise characteristics in NSFETs.

## 5. Conclusions

This study quantitatively analyzed the low-frequency noise characteristics of NSFET structures using TCADs. Evaluation of electric field distribution and trap formation behavior across different STI depths confirmed that as STI depth increases, the trapped charge density rises, leading to a corresponding increase in PSD. In contrast, shallower STI structures form fewer trapped charges near the channel sidewalls, thereby tending to suppress noise. Furthermore, replacing HfO_2_ with an HZO-based FE insulator improved gate–field control and reduced charge trapping, resulting in an overall enhanced noise performance. Optimization results demonstrated that the lowest-noise PSD was achieved with an STI depth of 3 nm and a SiO_2_/silicon interface trap density of 1 × 10^10^ eV^−1^cm^−2^. These findings indicate that complementary optimization of structural parameters (STI) and material parameters (interface traps and HZO) can improve the low-frequency noise characteristics of NSFETs. The results are expected to serve as important design guidelines for future low-noise, high-reliability nanoscale device design.

## Figures and Tables

**Table 1:** NSFET simulation parameters.

Parameter	Value
Channel length	14 nm
Nanosheet Width (N_W_)	22 nm
Nanosheet Thickness (N_T_)	7 nm
Phosphorus concentration (source, drain)	2 × 10^21^ cm^−3^
Boron concentration (channel)	1 × 10^15^ cm^−3^
Boron concentration (substrate)	2 × 10^18^ cm^−3^
HfO_2_	1 nm
TiN	1.5 nm
TaN	1.5 nm
TiAl	2 nm
SiO_2_	2 nm
Silicon	21.5 nm

**Table 2:** Simulated subthreshold swing and DIBL values for different STI depths.

STI Depth	SS (mV/dec)	DIBL (mV/V)
3 nm	72.38	−180.6
5 nm	72.33	−174.0
9 nm	63.8	−180.8

**Table 3:** LK equation parameters.

α (cm/F)	1.4 × 10^10^
Β (cm^5^/F/coul^2^)	−3.2 × 10^19^
γ (cm^9^/F/coul^4^)	1.2 × 10^28^

## Data Availability

The data presented in this study are available on request from the corresponding author.
